# Pulmonary rehabilitation following exacerbations of chronic obstructive pulmonary disease

**DOI:** 10.1590/S1516-31802009000500016

**Published:** 2010-02-03

**Authors:** Milo Puhan, Madlaina Scharplatz, Thierry Troosters, E. Haydn Walters, Johann Steurer, Cochrane Airways Group

## Abstract

**BACKGROUND:**

Pulmonary rehabilitation has become a cornerstone in the management of patients with stable Chronic Obstructive Pulmonary Disease (COPD). Systematic reviews have shown large and important clinical effects of pulmonary rehabilitation in these patients. In unstable COPD patients who have suffered from an exacerbation recently, however, the effects of pulmonary rehabilitation are less established.

**OBJECTIVES:**

To assess the effects of pulmonary rehabilitation after COPD exacerbations on future hospital admissions (primary outcome) and other patient-important outcomes (mortality, health-related quality of life and exercise capacity).

**SEARCH STRATEGY:**

Trials were identified from searches of CENTRAL, MEDLINE (Medical Literature Analysis and Retrieval System), EMBASE (Excerpta Medica Databases), PEDRO (Physiotherapy Evidence Database) and the Cochrane Central Register of Controlled Trials. Searches were current as of July 2008.

**SELECTION CRITERIA:**

Randomized controlled trials comparing pulmonary rehabilitation of any duration after exacerbation of COPD with conventional care. Pulmonary rehabilitation programmes needed to include at least physical exercise. Control groups received conventional community care without rehabilitation.

**DATA COLLECTION AND ANALYSIS:**

We calculated pooled odds ratios and weighted mean differences (WMD) using fixed-effects models. We requested missing data from the authors of the primary studies.

**MAIN RESULTS:**

We identified six trials including 219 patients. Pulmonary rehabilitation significantly reduced hospital admissions (pooled odds ratio 0.13 [95% confidence interval, CI 0.04 to 0.35], number needed to treat (NNT) 3 [95% CI 2 to 4], over 34 weeks) and mortality (pooled odds ratio 0.29 [95% CI 0.10 to 0.84], NNT 6 [95% CI 5 to 30] over 107 weeks). Effects of pulmonary rehabilitation on health-related quality of life were well above the minimal important difference (weighted mean differences for dyspnea, fatigue, emotional function and mastery domains of the Chronic Respiratory Questionnaire between 1.15 (95% CI: 0.94, 1.36) and 1.88 (95% CI:1.67, 2.09) and between -9.9 (95% CI:-18.05, -1.73) and -17.1 (95% CI: -23.55, -10.68) for total, impact and activity limitation domains of the St. Georges Respiratory Questionnaire). In all trials, pulmonary rehabilitation improved exercise capacity (60-215 meters in six-minute or shuttle walk tests). No adverse events were reported (two studies).

**AUTHORS’ CONCLUSIONS:**

Evidence from small studies of moderate methodological quality suggests that pulmonary rehabilitation is a highly effective and safe intervention to reduce hospital admissions and mortality and to improve health-related quality of life in COPD patients after suffering an exacerbation.

FURTHER INFORMATION:

Centro Cochrane do Brasil

Rua Pedro de Toledo, 598

Vila Clementino - São Paulo (SP) - Brasil

CEP 04039-001

Tel. (+55 11) 5579-0469/5575-2970

http://www.centrocohranedobrasil.org.br/

